# The therapeutic potential of descending regulatory pathways from brain to bone in osteoporosis: focusing on the brain-bone axis

**DOI:** 10.3389/fendo.2026.1750464

**Published:** 2026-03-04

**Authors:** Yi Rong, Lu Zhang, Maoting Xu, Yanan Chen, Xiaoxue Wang, Sheng Li, Guoqiang Yang, Guiquan Chen

**Affiliations:** Acupuncture and Rehabilitation Department, The Affiliated Traditional Chinese Medicine Hospital, Southwest Medical University, Luzhou, Sichuan, China

**Keywords:** bone fragility, brain-bone axis, interrelationship, neuroendocrine, osteoporosis

## Abstract

Osteoporosis (OP) is a growing global health concern, characterized by reduced bone mass, deterioration of bone microarchitecture, and consequently increased bone fragility. The brain-bone axis, a complex regulatory network encompassing the nervous, endocrine, and immune systems, elucidates the central role of the brain in regulating bone homeostasis. Consequently, this axis has become a major focus of interdisciplinary research into the pathogenesis of OP. However, the current understanding of the descending regulatory pathways from the brain to the bone remains incomplete. Therefore, this paper preliminarily explores the mechanisms and experimental evidence of different descending regulatory pathways from a new perspective. It integrates multiple descending regulatory pathways, discusses some of their interrelationships, and reveals the complex network nature of central bone metabolism regulation. Our objective is to elucidate the role of the central nervous system (CNS) in OP pathogenesis, thereby offering new insights and directions for future research on its prevention and treatment.

## 1 Introduction

Osteoporosis (OP) is a systemic metabolic bone disease characterized by reduced bone mass, impaired bone microarchitecture, and increased bone fragility (1). The fundamental mechanism involves an imbalance between osteoblast-mediated bone formation and osteoclast-mediated bone resorption. This imbalance, characterized by a relative predominance of resorption over formation, ultimately leads to bone loss. Postmenopausal osteoporosis (PMOP), a major subtype of primary OP, is primarily driven by the rapid decline in estrogen levels during menopause. The most significant bone loss typically occurs within the first 2–3 years following the onset of menopause. The global burden of OP is escalating in tandem with population aging. In China, the direct medical costs attributable to osteoporotic fractures are projected to reach $18.9 billion by 2035, with this Figure likely rising by approximately 34% when it comes to 2050 (2). OP poses a substantial societal burden and profoundly compromises the quality of life, especially in women. Consequently, the identification of novel therapeutic targets is of paramount importance. In this context, emerging evidence of a close relationship between the central nervous system (CNS) and bone metabolism offers promising new directions and insights for the OP prevention and treatment (3).

The brain-bone axis is an emerging research paradigm that describes a complex bidirectional regulatory network connecting the CNS and the bone. This axis integrates components ranging from specific brain structures [e.g., the cerebral cortex, hypothalamus, and autonomic nervous system (ANS)] to a multitude of signaling molecules, including neuroendocrine factors and hormones. Ultimately, it functions as the principal pathway through which the nervous system centrally regulates the balance between osteoblast-mediated bone formation and osteoclast-mediated bone resorption, thereby maintaining skeletal homeostasis (3, 4). The concept of the brain-bone axis underscores the critical role of the CNS in OP, presenting promising new avenues for identifying therapeutic targets and developing innovative prevention strategies.

**Figure 1:**
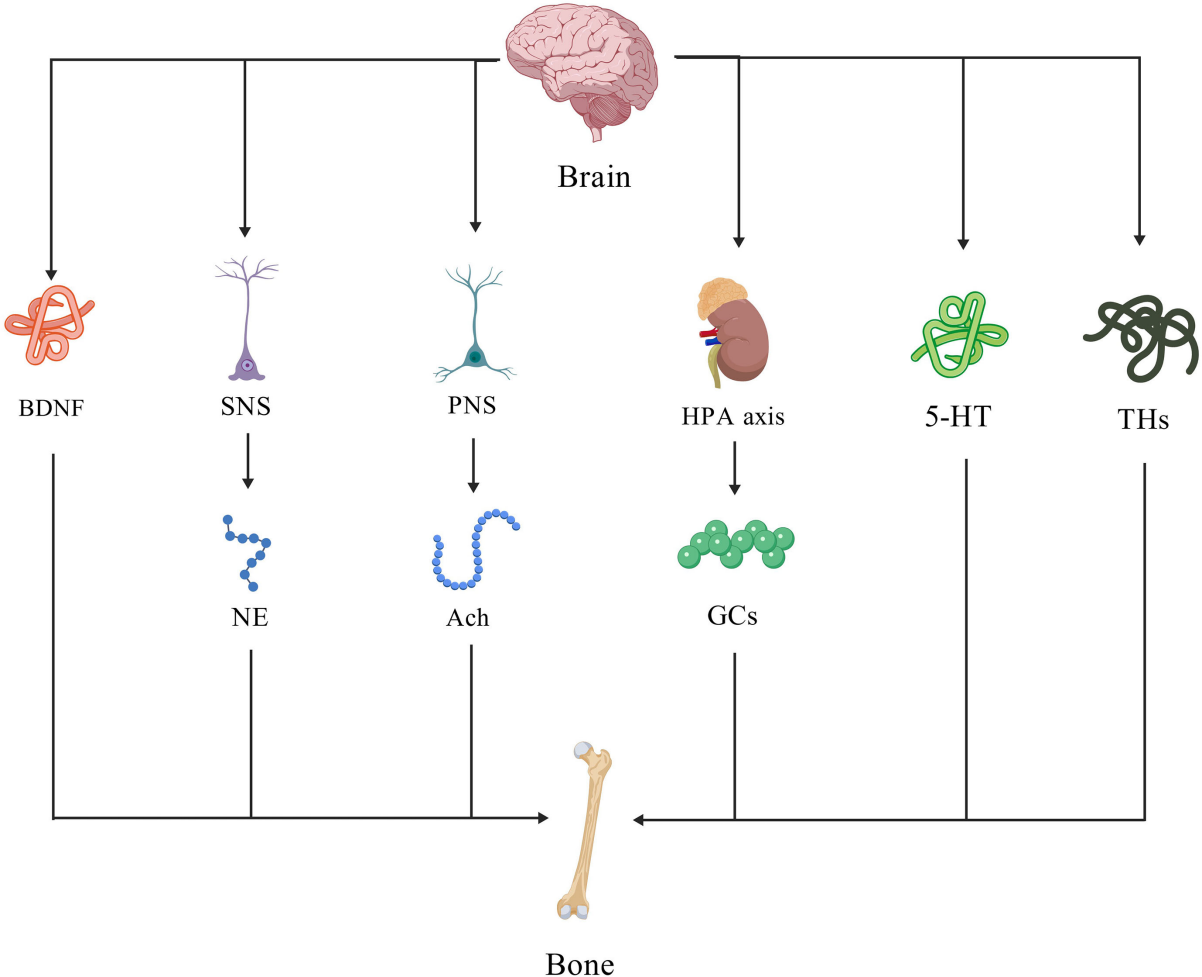
Overview of the descending regulatory pathways discussed in this review.

Based on recent literature, this review summarizes the current understanding of the brain-bone axis, centering on three key aspects: the mechanisms underlying key signaling molecules, and the direct regulation of several bone via neural pathways, (**Figure 1**). All Figure ures in this review were created using BioGDP.com (5).

## 2 Downstream regulation from the CNS to the bone

### 2.1 Brain-derived neurotrophic factor

Brain-derived neurotrophic factor (BDNF) is the most abundant, widely distributed, and extensively characterized neurotrophic factor in the mammalian CNS. In rodents, its expression is predominantly localized to regions such as the hippocampus and entorhinal cortex. The complexity of BDNF expression is underscored by its transcriptome, which is driven by at least nine distinct promoters that generate over 20 transcript variants. Notably, all these splice variants encode an identical precursor protein, known as pre-proBDNF (6). Functioning as an autocrine and paracrine factor, BDNF exerts its effects by binding primarily to its high-affinity receptor, tropomyosin receptor kinase B (TrkB), on pre- and postsynaptic membranes. Through this signaling pathway, BDNF is critical for regulating neuronal survival, differentiation, growth, and the maintenance of synaptic plasticity and normal neuronal function (7). Recent studies have provided evidence that serum BDNF levels are positively correlated with bone mineral density (BMD) in female athletes (8). In elderly women, a daily step count exceeding 5,000 has been associated with elevated levels of oxidative stress markers (e.g., GSH and MDA) and significant alterations in bone turnover markers (e.g., osteocalcin and CTX-1) in blood samples. Notably, this level of physical activity is also linked to a marked increase in circulating BDNF levels (9). Collectively, these findings suggest that BDNF levels may serve as a potential indicator of skeletal health and point to a possible causal role of BDNF in bone metabolism.

**Figure 2:**
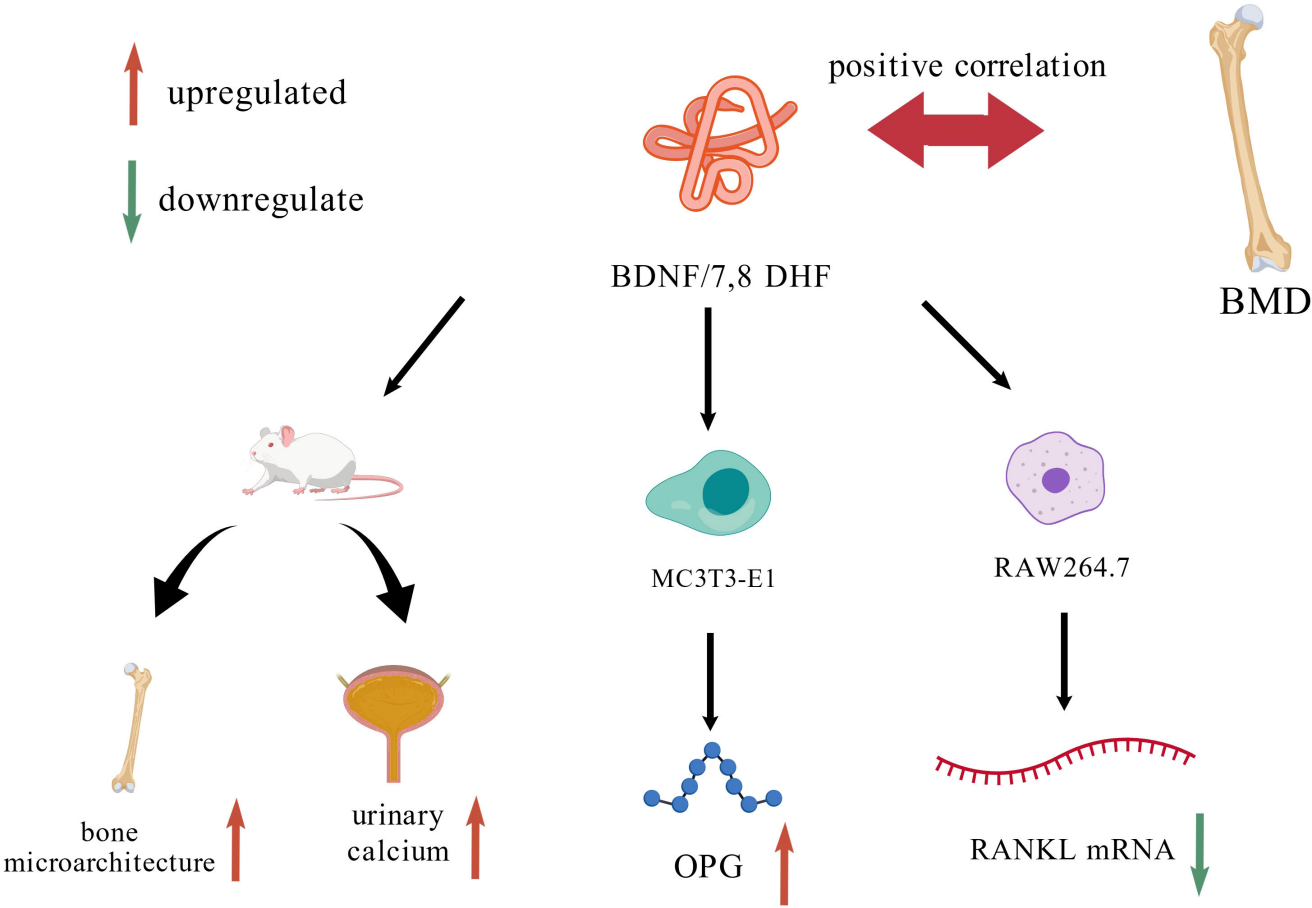
Regulatory mechanisms of BDNF on bone tissue and associated cells.

To address these questions, 7,8-dihydroxyflavone (7,8-DHF), a small-molecule BDNF mimetic, has been widely utilized in basic research. 7,8-DHF specifically binds to the extracellular domain of the TrkB receptor with high affinity, crosses the blood-brain barrier (BBB), and activates downstream signaling pathways such as MAPK and Akt, thereby mimicking the neurotrophic effects of BDNF (10, 11). In the ovariectomized (OVX) rat model, it was found that 7,8-DHF significantly improved bone microstructure and biomechanical properties while reducing urinary calcium levels, without exhibiting any uterotrophic activity. (12). At the cellular level, 7,8-DHF promotes bone formation by enhancing the differentiation and mineralization capacity of osteoblast precursor cells (e.g., MC3T3-E1) and stimulating the secretion of osteoprotegerin (OPG). Concurrently, it inhibits osteoclastogenesis by suppressing RANKL-induced osteoclast differentiation in precursor cells (e.g., RAW264.7) and downregulating RANKL mRNA expression. This dual action on both anabolic and catabolic pathways effectively modulates bone remodeling balance (**Figure 2**). The pro-osteogenic effects are potentially mediated through the activation of key signaling pathways, including cAMP-response element binding protein (CREB) and Wnt/β-catenin (12, 13).

### 2.2 Sympathetic nervous system pathways and norepinephrine

The sympathetic nervous system (SNS), one of the two primary divisions of the ANS, originates from the intermediolateral nucleus of the spinal cord’s gray matter in the thoracic and upper lumbar (T1-L2) regions. The peripheral ganglia of the SNS comprise the paravertebral chain ganglia, which flank the vertebral column, and the prevertebral ganglia, located anterior to the aorta in the abdomen. Cholinergic preganglionic neurons connect the CNS to these ganglia. Noradrenergic postganglionic neurons then project from the ganglia to innervate various target tissues, including the heart, digestive system, and reproductive organs. It is noteworthy that bone tissue receives direct sympathetic innervation, primarily via the periarterial plexuses (14, 15). Anatomical tracing studies published over two decades ago have demonstrated that sensory nerves from the femur project to the dorsal root ganglia (DRG) at corresponding lumbar spinal levels. Furthermore, these neural pathways were shown to extend to higher brain centers, including the anterior cingulate cortex, motor cortex, paraventricular nucleus (PVN) of the hypothalamus, and specific nuclei within the brainstem (16, 17). The SNS mediates its effects primarily via the release of norepinephrine (NE), which activates adrenergic receptors (ARs) on target cells. Consequently, circulating NE levels are widely used as a key biomarker of overall sympathetic tone (18). Studies have demonstrated that tibial NE levels are significantly elevated in ovariectomized (OVX) mice compared to sham-operated controls. *In vitro*, treatment with exogenous NE induces phosphorylation of the transcription factor CREB in osteoclasts. This, in turn, promotes the secretion of extracellular vesicles (EVs) by osteoclasts, ultimately enhancing bone resorptive activity (19). These findings suggest that β-ARs on bone cells represent a critical signaling intermediary for sympathetic regulation of bone metabolism, operating alongside direct neural innervation.

β-ARs belong to the G-protein-coupled receptor (GPCR) superfamily. Upon binding NE, the primary sympathetic neurotransmitter, β-ARs activate stimulatory G (Gs) proteins. This, in turn, triggers adenylate cyclase activity, leading to intracellular accumulation of cyclic AMP (cAMP) and propagation of the signal from the membrane to the cytoplasm (20). Pharmacological blockade of β-ARs has been shown to significantly improve tibial trabecular bone microarchitecture in ovariectomized mice and to accelerate the process of fracture healing (21, 22). Consistent with these *in vivo* observations, *in vitro* experiments revealed that β-AR activation inhibits osteogenic differentiation of bone marrow-derived mesenchymal stem cells (BMSCs) and simultaneously promotes osteoclastogenesis from bone marrow macrophages (BMMs). This demonstrates that the NE-β-AR signaling pathway directly regulates bone remodeling by dually suppressing bone formation and enhancing bone resorption at the cellular level (22). Intriguingly, differentiated mature osteoblasts, but not osteoclasts, express the NE transporter (NET), which functions to clear extracellular NE and terminate its signaling. Corroborating this functional role, pharmacological inhibition of NET in wild-type mice results in a low bone mass phenotype, likely due to impaired NE clearance and subsequent overactivation of β-adrenergic signaling (23).

In summary, SNS regulates bone remodeling through two principal pathways: direct neural innervation and systemic NE signaling. Accumulated evidence indicates that sympathetic tone generally exerts a catabolic effect on the skeleton, suppressing bone formation and potentiating bone resorption.

### 2.3 The parasympathetic nervous system and acetylcholine

The parasympathetic nervous system (PNS) constitutes the other major division of the ANS, with the vagus nerve (cranial nerve X) representing its most prominent and widely distributed component. Originating in the medulla oblongata, the vagus nerve projects extensively to innervate numerous thoracic and abdominal organs, including the heart, lungs, liver, and gastrointestinal tract. It plays a critical role in the regulation of essential physiological functions such as heart rate, respiration, and immune responses (24). As a major cholinergic nerve, the vagus nerve mediates critical functions—including trophic, anti-inflammatory, and analgesic effects—primarily through the release of its principal neurotransmitter, acetylcholine (ACh). Growing evidence underscores its significant role in the pathophysiology of various musculoskeletal disorders, such as rheumatoid arthritis, osteoarthritis, and spondyloarthritis (25). Notably, intraoperative vagus nerve stimulation (VNS) has been shown to significantly increase lumbar spine bone mineral density (BMD) in patients. This finding reveals the therapeutic potential of modulating parasympathetic activity and opens new avenues for developing innovative diagnostic and treatment strategies for skeletal disorders, especially OP (26).

Similar to the relationship between SNS and NE, PNS’s primary neurotransmitter, acetylcholine (ACh) also plays a crucial role in regulating bone remodeling (27). ACh is a principal neurotransmitter in vertebrates and the key signaling molecule in PNS postganglionic nerve fibers. It is synthesized within nerve terminals through a reaction between acetyl-CoA and choline, catalyzed by the enzyme choline acetyltransferase (28). The primary classes of acetylcholine receptors are nicotinic (nAChRs) and muscarinic (mAChRs). The abundant expression of mAChRs in bone tissue implies a significant role for the PNS in regulating bone remodeling. mAChRs consist of five subtypes: the excitatory M1, M3, and M5, and the inhibitory M2 and M4. Their distribution patterns within the skeleton are both species- and site-specific (28, 29). Female mice with M3 receptor knockdown displayed significantly lower femoral bone density, impaired biomechanical properties, and markedly suppressed bone formation compared to wild-type controls (30). Furthermore, brain-derived ACh positively regulates the maintenance of peak bone mass in mice, with the effect exhibiting sexual dimorphism. This observed dimorphism may be attributable to the subtype-specific distribution of mAChRs within skeletal tissue (31). nAChRs also play a critical role in bone remodeling. These receptors function as pentameric ligand-gated ion channels composed of various combinations of subunits, such as α1-α10, β1-β4, and others (32). Early studies reported that stimulation of mouse BMMs with nAChR agonists—both non-selective and α7-subtype-specific—produces a bidirectional, dose-dependent effect on osteoclastogenesis: inhibition at higher concentrations but potentiation at lower doses. *In vitro* experiments further confirmed that nAChR ligands can inhibit the differentiation of osteoclast precursors (33). Genetic deletion of the nAChR α9 subunit in mice results in significant deterioration of bone strength, microarchitecture, and trabecular morphology (34). Interestingly, some reports indicate that α7 knockout mice exhibit no significant differences in bone volume fraction or trabecular morphological parameters compared to wild-type mice. At 6 weeks of age, mRNA expression of α1, α4, α5, and α9 in tibial tissue was significantly higher than in 3-week-old mice, suggesting that α9 may play a primary role in promoting bone formation (35, 36). Additionally, the activation of the anti-inflammatory pathway mediated byα7 nAChRs and the upregulation of estrogen receptor (ERs) expression may play important roles in regulating bone mass. Rats treated with the α7-nAChRs agonist PNU-282987 exhibited a significant increase in ERs expression and bone mineral density, along with a blockade of NF-κB nuclear translocation and a marked suppression in the synthesis and release of multiple pro-inflammatory cytokines, including TNF-α, IL-1β, and IL-6. Notably, TNF-α and IL-6 are crucial stimulatory factors for osteoclastogenesis, directly impacting the proliferation and differentiation of osteoclasts (37–40).

**Figure 3:**
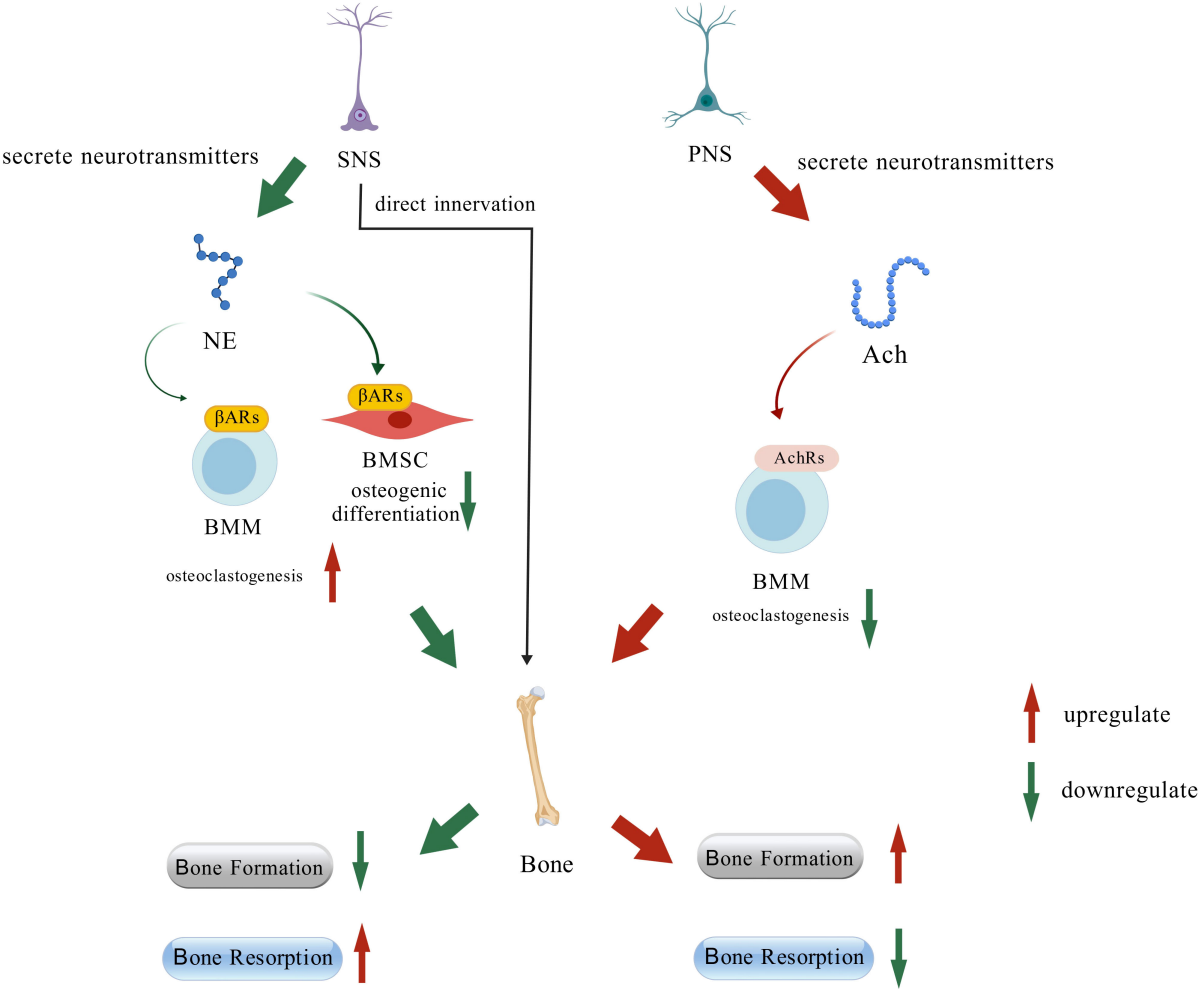
Regulation of the bone tissue via the ANS pathway.

Similar to the SNS, the PNS modulates bone remodeling via neurohumoral signaling. A key distinction, however, PNS exerts a more positive effect on osteogenesis by modulating ERs and the anti-inflammatory pathway.in contrast to the catabolic influence of the SNS (**Figure 3**).

### 2.4 The hypothalamic-pituitary-adrenal axis and glucocorticoids

Glucocorticoids (GCs) are a major class of steroid hormones that play critical roles in a wide range of physiological processes, such as metabolism, immune and inflammatory responses, development, and reproduction (41). The production of GCs depends on the hypothalamic-pituitary-adrenal (HPA) axis. This system includes the paraventricular nucleus of the hypothalamus, the pituitary gland, and the adrenal cortex, connecting the CNS to peripheral organs. When the HPA axis is activated, the hypothalamus releases corticotropin-releasing hormone (CRH), which stimulates the pituitary gland to secrete adrenocorticotropic hormone (ACTH). ACTH then promotes the synthesis and release of GCs from the adrenal glands. Finally, GCs enter the circulation to regulate the function of various organs and tissues, including feedback effects on the brain (42, 43).

The link between GCs and bone loss is sufficiently strong that a major category of secondary OP is termed glucocorticoid-induced osteoporosis (GIO). This condition is characterized by a profound imbalance in bone remodeling, typically manifesting as a rapid increase in bone resorption coupled with a suppression of bone formation in patients undergoing exogenous GC therapy (44, 45). Whether endogenous or exogenous GCs, a substantial body of research has demonstrated their effects on various bone cells, including osteoblasts, osteoclasts, and osteocytes (46–48). Firstly, Treatment with GCs in both human osteoblast culture medium and Wnt3a-conditioned medium derived from human osteoblasts resulted in decreased T-cell factor (Tcf)/lymphoid enhancer factor (Lef) transcriptional activity, along with reduced intracellular accumulation and nuclear translocation of β-catenin. The canonical Wnt inhibitor DKK1 was able to recapitulate these effects. This similarity strongly suggests that the inhibition of osteogenesis and upregulation of intracellular RANKL by GCs are mediated through suppression of the Wnt signaling pathway (49, 50). Furthermore, GCs induce a dose-dependent reduction in Wnt16 expression in both MC3T3-E1 cells and mouse BMSCs. Consistent with this cellular effect, GC administration also leads to a decrease in BMD in mice (51). Additionally, GCs promote bone resorption through multiple mechanisms. They enhance the collagenase-mediated degradation of type I collagen by osteoblasts in the extracellular matrix. Furthermore, GCs can induce osteoblast autophagy and apoptosis, which also contributes to the net loss of bone (52–54). Tracing back based on the above clues, it can be found that in both human and mouse bone tissues, 11β-hydroxysteroid dehydrogenase type 1 (11β-HSD1) plays a critical role in the regulation of GCs and osteoblasts.

11β-HSD1 is an enzyme heavily involved in prereceptor GCs metabolism. Its expression in bone increases with age, and it catalyzes the reduction of inactive GCs to their active state for receptor binding. Its function is likely closely associated with metabolic syndromes such as type II diabetes and central obesity (55). In mouse-derived cells, it has been observed that 11β-HSD1 can inhibit osteogenic differentiation, and this effect can be reversed by the 11β-HSD1 inhibitor KR-67500 (56). Furthermore, it was observed that in bone marrow-derived human mesenchymal progenitor cells (hMSCs) overexpressing 11β-HSD1, adipogenic differentiation was significantly increased, while osteogenic differentiation was inhibited (57). This mechanism was also demonstrated in the clinical trial by Abbas et al.: PMOP patients who orally took the 11β-HSD1 inhibitor AZD4017 showed lower serum 11β-HSD1 levels and relatively increased osteocalcin levels compared to the placebo group. However, it could not fully reverse the bone loss caused by estrogen deficiency (58).

Studies in model organisms such as zebrafish confirm the evolutionarily conserved effects of GCs. In zebrafish, GC treatment reduces the activity of the osteoblast metabolic enzyme alkaline phosphatase (ALP) and downregulates key osteogenic marker genes, including RUNX2 and SP7 (59). Collectively, the evidence demonstrates that GCs suppress osteogenesis through a multitude of conserved mechanisms across multiple species.

Through their action on osteoblasts, GCs increase the RANKL/OPG ratio, thereby indirectly promoting osteoclast differentiation and maturation. However, the direct effects of GCs on osteoclasts themselves are more complex and not purely stimulatory (47). Early research indicated that GCs inhibit the proliferation of osteoclast precursors (BMMs) without impairing their differentiation into mature osteoclasts. Furthermore, GCs were found to suppress the bone-resorbing activity of mature osteoclasts while simultaneously prolonging their survival by inhibiting apoptosis (60). Simultaneously, GCs upregulate calcitonin receptor (CTR) mRNA expression in mouse osteoclast-like cells (OCLs), further inhibiting bone resorption (61). However, contemporaneous studies also revealed that GCs can promote osteoclastogenesis by significantly downregulating the endogenous production of interferon-β (IFN-β). As IFN-β is a known inhibitor of RANKL signaling, its reduction removes a key endogenous brake on osteoclast differentiation (62). Recent studies reveal a species-specific dual role for GCs. In mice, GCs promote the proliferation of classical monocytes and their differentiation into osteoclasts. Conversely, in bluefin tuna, GCs inhibit bone repair by preventing the accumulation of both osteoclasts and osteoblasts at fracture sites (63, 64). Crucially, in genetically modified mice lacking the glucocorticoid receptor (GR) specifically in osteoclasts, the inhibitory effect of GCs on bone formation was significantly attenuated. This finding provides direct genetic evidence that osteoclasts are essential cellular mediators of GC-induced suppression of osteogenesis (60). The evidence summarized above indicates that the effects of GCs on osteoclasts are complex and bidirectional. The precise mechanisms underlying these context-dependent effects remain an important area for future investigation.

**Figure 4:**
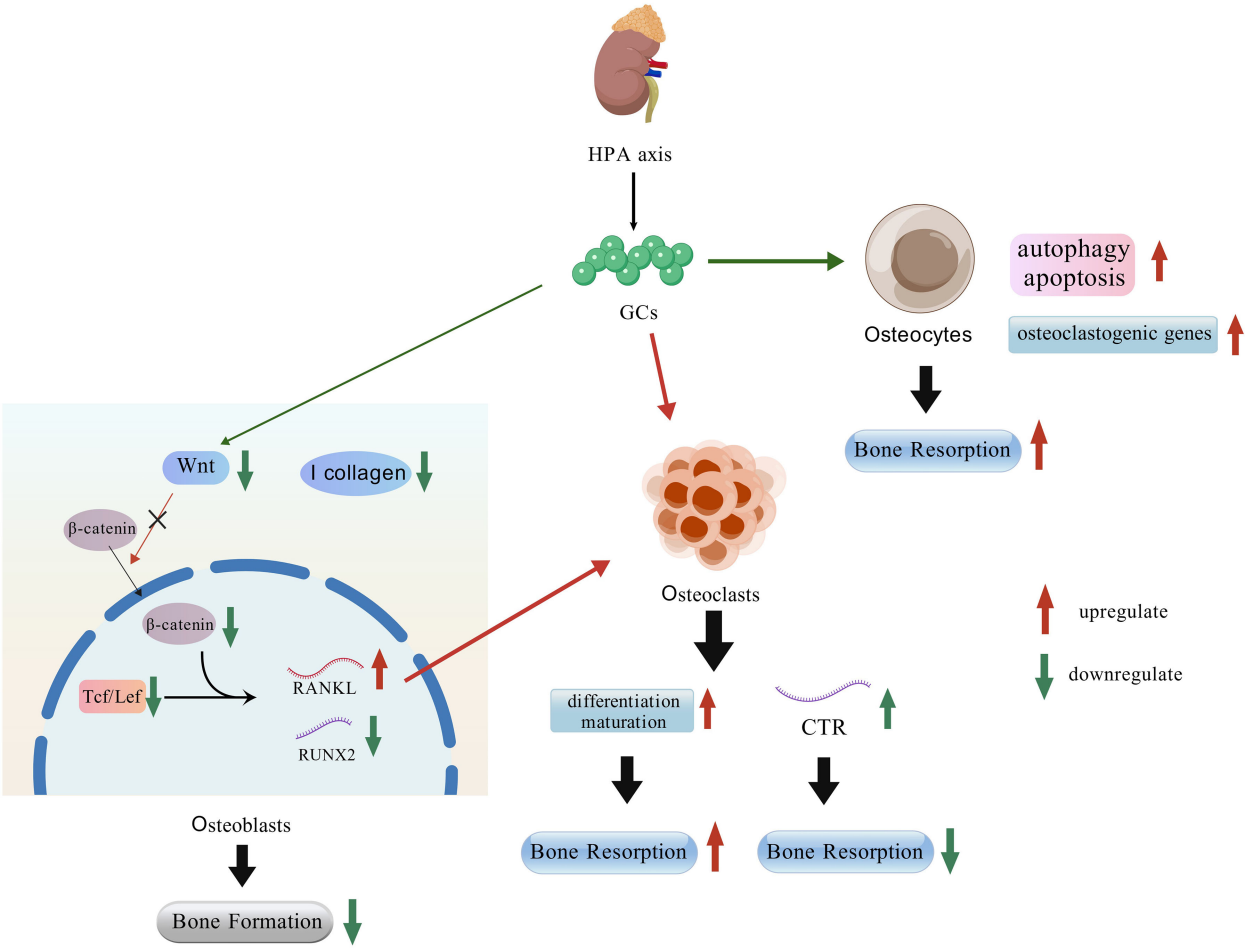
Regulation of bone by the HPA axis and GCs.

Another critical aspect of GCs’ skeletal effects centers on osteocytes. As the terminal differentiation stage of osteoblasts, osteocytes represent over 90% of all bone cells and play a central role in regulating bone aging and maintaining skeletal homeostasis (65). Preliminary histological studies revealed enlarged osteocyte lacunae specifically in the lumbar vertebrae of GC-treated mice. This specific morphological change was not observed in mice with estrogen deficiency alone, suggesting it may be a distinct pathological feature of GC-induced bone deterioration (66). *In vitro* experiments demonstrated that GC treatment of mouse osteocytes significantly reduced cell viability, increased autophagy markers, and promoted autophagosome accumulation. These findings were corroborated by *in vivo* studies, collectively indicating that GCs induce osteocyte autophagy and apoptosis (67, 68). The regulation of autophagy and apoptosis by GCs is not mutually exclusive but exhibits a dose-dependent relationship. For instance, in the MLO-Y4 osteocyte cell line, low-dose exogenous GCs primarily induce autophagy, whereas high doses trigger apoptosis (69). At the molecular level, GCs upregulate the expression of key osteoclastogenic genes—such as Acp5 (TRAP), Mmp13, Atp6v0d2, and Ctsk—in osteocytes. This transcriptional reprogramming of osteocytes contributes to the promotion of cortical bone resorption (70). Although research on the effects of GCs on osteocytes remains limited, the existing body of evidence consistently points toward a net catabolic effect, primarily driven by enhanced bone resorption (**Figure 4**).

### 2.5 Serotonin

Serotonin, or 5-hydroxytryptamine (5-HT), is a major monoamine neurotransmitter derived from tryptophan. It is widely distributed in the CNS and the gastrointestinal tract, where it regulates critical functions including mood, cognition, pain perception, sleep, memory, and gut motility (71, 72). The pleiotropic effects of serotonin (5-HT) are mediated by its cognate receptors (5-HTRs). These receptors are categorized into seven families based on their distinct signaling pathways. As members of the G protein-coupled receptor (GPCR) superfamily, 5-HTRs share a characteristic structure consisting of seven transmembrane-spanning helices, with an extracellular N-terminus and an intracellular C-terminus (73). Ligand binding to GPCRs induces a conformational change in the transmembrane domain, initiating intracellular signal transduction. This process involves the activation of heterotrimeric G proteins and G protein-coupled receptor kinases (GRKs), which propagate the extracellular signal into the cell and trigger a downstream signaling cascade (74). 5-HT is synthesized directly from the amino acid tryptophan. Although the biosynthetic pathway is similar in the brain and the gut, the maintenance of cerebral 5-HT synthesis—which depends on adequate tryptophan availability—requires normal HPA axis function (75). Both gut-derived and brain-derived 5-HT regulate bone formation, although their specific effects on bone mass can be divergent. Overall, central 5-HT signaling appears to exert a more dominant influence on the regulation of bone mass (76, 77). This review will focus specifically on the role of brain-derived 5-HT in the regulation of bone mass.

The seminal work by Yadav et al. showed that leptin-deficient mice exhibit elevated brain 5-HT levels and high bone mass, phenotypes reversible upon genetic suppression of tryptophan hydroxylase 2 (Tph2). Their research established that brain-derived 5-HT regulates bone mass through the ventromedial hypothalamus (VMH) by attenuating SNS tone. Mechanistically, 5-HT signaling in the VMH enhances the expression and phosphorylation of the transcription factor CREB, which modulates the transcription of key genes controlling sympathetic outflow. The net effect of this central regulatory circuit is a reduction in sympathetic tone, leading to increased bone formation and bone mass (77, 78). Collectively, these findings establish 5-HT as a central regulator of bone metabolism. Its effects are mediated downstream through the ANS and NE signaling pathways. Notably, these pathways exhibit partial synergy and crosstalk with BDNF-mediated signaling. The clinical relevance of this mechanism is supported by the observation that patients with neuropsychiatric disorders characterized by central 5-HT deficiency frequently present with comorbid OP and an elevated risk of fragility fractures (79, 80).

**Figure 5:**
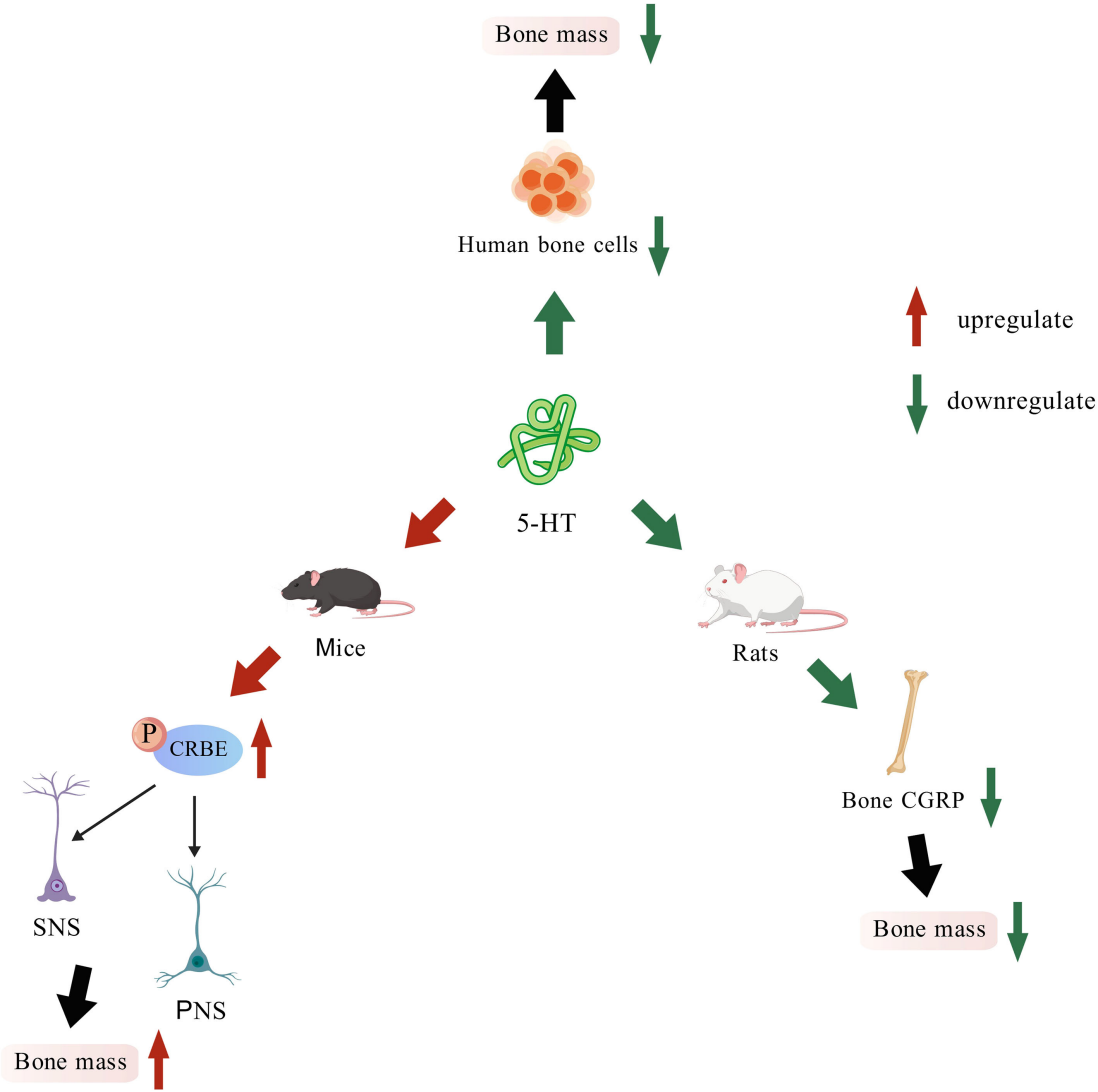
Regulation of bone mass by 5-HT across different species.

In ovariectomized (OVX) rats, levels of 5-HT and its mRNA are significantly elevated in the spinal cord. This elevation may be linked to pain-induced release of calcitonin gene-related peptide (CGRP) in the spinal dorsal horn of OVX rats. It has been proposed that spinal CGRP promotes 5-HT release, which in turn downregulates CGRP expression within bone tissue. This paradoxical feedback loop may ultimately contribute to accelerated bone loss (81). Studies by Lam et al. reported a related phenomenon: in a rat model of depression treated solely with selective 5-HT reuptake inhibitors (SSRIs), no significant bone loss was observed. In contrast, *in vitro* evidence demonstrates that SSRIs can directly induce apoptosis and suppress function in both human osteoblasts and osteoclasts (82, 83) (**Figure 5**). These seemingly paradoxical phenomena can be attributed to the functional antagonism between brain-derived and gut-derived serotonin. Since 5-HT cannot cross the blood-brain barrier, there are notable differences in both the production and function of brain-derived and gut-derived 5-HT. Gut-derived 5-HT, primarily secreted by enterochromaffin cells, acts on the 5-HT_1B_ receptors of osteoblasts, inhibiting their proliferation and function. Interestingly, the effector molecule in the intermediate cascade of reactions remains CREB (84).

### 2.6 Thyroid hormone

Thyroid hormones (THs), secreted by thyroid follicular cells, are critical regulators of metabolism, growth, and development. Their biosynthesis is governed by the hypothalamic-pituitary-thyroid (HPT) axis and depends on the availability of inorganic iodide. The enzyme thyroid peroxidase (TPO) catalyzes two key steps: the iodination of tyrosine residues on thyroglobulin (TG) and the coupling of iodotyrosines to form thyroxine (T4) and triiodothyronine (T3). The entire process is under negative feedback control, primarily by thyroid hormones themselves, which suppress the release of thyrotropin-releasing hormone (TRH) from the hypothalamus and thyroid-stimulating hormone (TSH) from the pituitary, maintaining systemic homeostasis (85). The biological actions of THs are primarily mediated by thyroid hormone receptors (THRs), which are encoded by two distinct genes: THRA (TRα) and THRB (TRβ) (86). The primary thyroid hormone secreted into the circulation is thyroxine (T4), which functions as a prohormone. T4 is activated by outer-ring deiodination to form the biologically active hormone T3. T3 then binds to nuclear THRs to modulate the transcription of target genes (87). The expression of THRs and specific transporters in chondrocytes, osteoblasts, and osteoclasts provides the cellular basis for T3 action on the skeleton. Mechanistically, T3 exerts its anabolic effects primarily by activating the IGF-1 and Wnt/β-catenin signaling pathways (88–90). Beyond these core pathways, THs employ additional mechanisms to regulate bone homeostasis in a cell- and tissue-specific manner.

Genetic disruption of TH signaling in mice results in severe impairment of endochondral ossification. Conversely, exogenous TH administration rescues this phenotype, as evidenced by the restoration of normal chondrocyte differentiation and the expression of key markers including collagen II, collagen X, and osteocalcin (91). This phenomenon is also widely observed in humans. For example, children with hypothyroidism, which leads to decreased levels of THs, typically present with severe defects in endochondral ossification, resulting in bone dysplasia (88). In adults, THs have been shown to significantly increase serum ALP activity and maintain normal osteoblast function. However, both excessively high and low levels of THs can lead to bone loss through different mechanisms, which may be related to the inhibitory effects of THs on osteoclast activity and osteoblast proliferation (92, 93). Furthermore, in an experiment using chondrocytes derived from fertilized eggs, it was observed that THs promote the upregulation of intracellular bone morphogenetic protein 4 (BMP-4) expression and elevate collagen X mRNA levels (94). Perhaps the regulatory mechanisms of THs on bones also exhibit certain species specificity.

Additionally, in TH-specific transporter monocarboxylate transporter 10 (MCT10) knockout mice, a reduction in femoral trabecular bone volume accompanied by decreased osteoblast numbers was observed at 12 weeks, confirming that THs also exert regulatory effects on osteoblasts (95). T3 regulates osteoblast differentiation and mineralization primarily through the IGF-1 and Wnt/β-catenin signaling pathways. However, its overall effects are highly context-dependent, exhibiting significant species specificity as well as variable outcomes—ranging from promotion to inhibition to no effect—on the proliferation of different osteoblast cell lines depending on their origin and passage number (88). For example, T3 inhibits the growth of osteoblasts derived from mouse skull bone but promotes the proliferation of human osteoblast-like cells (96, 97).

However, reports indicate that excessive levels of THs may lead to bone loss by increasing the activity and number of osteoclasts (98, 99). But recent *in vitro* studies show that THs inhibit osteoclast differentiation from mouse macrophage precursors by activating the AMPK pathway, thereby helping preserve bone mass. Knocking out THRs reverses this effect (100). Although THs modulate the Wnt/β-catenin pathway in osteoclasts, research confirms that the effects of T3 on these cells are independent of the downstream RANKL signaling cascade (98). Therefore, the direct effect of THs on bone resorption remains unclear at present.

In summary, the regulation of bone cells by THs is complex and may even be bidirectional or antagonistic. Based on existing research, potential confounding factors likely include cell lines, passage numbers, cell types, species, and the developmental stage of primary cells.

## 3 Discussion

Bone is not an isolated organ. Therefore, a comprehensive understanding of OP pathogenesis and the development of effective treatments requires moving beyond a narrow focus on bone cells alone. Compelling evidence now establishes the brain as a critical regulator in the initiation and progression of this disease. This review synthesizes current evidence for the brain’s descending regulation of the skeleton, establishing bone as a target organ of the CNS. Major pathways identified include BDNF, the ANS (comprising the SNS and PNS), the HPA axis, 5-HT, and THs. The BDNF and PNS pathways generally promote osteogenesis, whereas the SNS and HPA axis pathways exert inhibitory effects. Other pathways discussed exhibit more complex context-dependent or functional antagonism.

Critically, these pathways do not operate in isolation but form an integrated regulatory network. A prime example is the HPA axis and the ANS, which function as core components of the stress response system to coordinate adaptation to daily challenges. Their coordinated function is exemplified by the concurrent regulation of heart rate and the dynamic control of stress-induced cortisol secretion and recovery (101). When the stress response system becomes dysregulated, the resulting allostatic load inflicts cumulative damage on the brain and body, manifesting as hippocampal atrophy and bone loss. Estrogen appears to confer neuroprotective benefits that mitigate this damage. This pathophysiology underscores the integral role of the HPA axis and ANS dysregulation in the pathogenesis of OP. Specifically, this is manifested as the HPA axis and the ANS working synergistically in daily life to maintain stress balance, while under the influence of estrogen, they inhibit bone loss caused by allostatic load. (102). The ANS is modulated by cardiovascular reflexes—such as the baroreceptor and chemoreceptor reflexes—with the nucleus tractus solitarius (NTS) acting as the central integration site for these afferent signals. Experimental evidence confirms that 5-HT, by acting on receptors within the NTS, participates in regulating both the sympathetic and parasympathetic branches of the ANS. This mechanism reveals an indirect pathway through which central 5-HT signaling can modulate skeletal muscle function, complementing its potential direct effects (103, 104). Further evidence indicates that THs may influence bone, in part, through downstream ANS pathways. For example, the bone loss induced by TH excess in mice is attenuated by genetic deletion of αARs. Additionally, T3 has been shown to interact with 5-HT receptors to modulate hippocampal BDNF expression, suggesting potential crosstalk between endocrine and monoaminergic systems in the central regulation of bone (97, 105).The above evidence preliminarily suggests the existence of a complex regulatory network. That is, beyond the direct impact of each pathway on bone, both THs and 5-HT can transmit neural signals to the ANS by binding to receptors at different sites, thereby achieving indirect regulation of bone.

The preceding discussion outlined how BDNF, by activating the Wnt pathway in bone tissue, upregulates downstream effectors such as RUNX2 and OPG to promote bone formation. Notably, RUNX2—a central transcription factor in this osteogenic pathway—also functions as a key signaling molecule in the upstream bone-to-brain communication axis. RUNX2 is a master transcription factor governing skeletal development. Its functions include inducing chondrocyte proliferation, promoting the commitment of bone progenitor cells to the osteoblast lineage, and driving the subsequent maturation of pre-osteoblasts into functional osteoblasts (106). RUNX2 promoter activity is detected in the mouse brain, with particularly high levels in the hippocampus and no significant activity in the cerebellum. Genetic knockdown of RUNX2 significantly attenuates brain injury and edema following middle cerebral artery occlusion (107–109). This appears to suggest the existence of additional descending regulatory pathways with RUNX2 as the central hub molecule.

Clinical evidence confirms the link between CNS disorders (e.g., Alzheimer’s disease, anorexia nervosa) and OP. Key mechanisms include HPA axis hyperactivity and excessive SNS activation, which drive bone loss. Additionally, long-term use of exogenous GCs and TH is a common cause of iatrogenic OP (110, 111). The findings reviewed in this paper highlight the therapeutic potential of non-pharmacological interventions—such as physical exercise—for skeletal disorders. These benefits are mediated through multiple pathways: by improving emotional state and stress resilience to normalize ANS and HPA axis activity, by elevating central BDNF levels, and by inhibiting bone loss through downregulation of resorptive signaling networks.

This review has several limitations. First, the exploration of the downstream brain-to-bone regulatory network remains preliminary, with mechanistic insights into individual pathways requiring further depth. Second, the upstream bone-to-brain regulatory axis is not discussed in detail. Finally, the reliance on multi-species literature may introduce bias in interpreting the relative importance and conserved function of the various pathways described. In summary, the evidence presented delineates a brain-bone axis that functions as a highly integrated network engaging the nervous, endocrine, and immune systems. This framework provides a novel, multidisciplinary foundation for developing future strategies to prevent and treat skeletal metabolic disorders such as OP.
